# Weight bearing training alleviates muscle atrophy and pyroptosis of middle-aged rats

**DOI:** 10.3389/fendo.2023.1202686

**Published:** 2023-08-30

**Authors:** Pengyu Fu, Lijing Gong, Luyao Yang, Shuning Tang, Fangyuan Ma

**Affiliations:** ^1^ Department of Physical Education, Northwestern Polytechnical University, Xi’an, China; ^2^ Key Laboratory of Physical Fitness and Exercise of Ministry of Education, Beijing Sport University, Beijing, China; ^3^ College of Education, Zhejiang University, Hangzhou, China; ^4^ School of Public Health, Fudan University, Shanghai, China; ^5^ School of Life Sciences, Nankai University, Tianjin, China

**Keywords:** aging, muscle atrophy, obesity, weight-bearing training, pyroptosis

## Abstract

**Background:**

Age-related muscle atrophy and adipose accumulation begin to occur in young and middle-aged individuals, and exercise at an early age improves body composition. Pyroptosis may play an essential role in age-related low-grade inflammation. This study aimed to explore the alleviation of muscle atrophy by weight-bearing training with increasing age *via* inhibition of pyroptosis.

**Methods:**

Ninety 8-month-old male SD rats were randomly divided into three groups: (1) normal baseline group (N group, *n* = 10), sacrificed after adaptive feeding; control group (C group, *n* = 40); and weight-bearing running group (R group, *n* = 40). Blood samples, adipose tissue (AT), and extensor digitorum longus (EDL) were collected after 8, 16, 24, and 32-weeks intervention.

**Results:**

The body weight, muscle mass, fat mass, plasma lipid, AT wet weight, adipocyte cross-sectional area (CSA), and apoptosis rates of AT and EDL were increased, while the muscle mass, wet weight, and fiber CSA of EDL were decreased by aging, which were reversed by exercise. Weight-bearing training promoted protein synthesis in EDL, inhibited protein degradation in EDL, and expression of pyroptotic key proteins in EDL and AT in rats.

**Conclusion:**

Weight-bearing training improves body composition and alleviates age-related muscle atrophy in rats, and its mechanism may be related to the inhibition of pyroptosis in the EDL and AT and the improvement of muscle protein metabolism.

## 1 Introduction

According to the statistics of the World Health Organization, by 2050, the total number of elderly people over the age of 65 in the world will exceed 2 billion (2003) (1). Skeletal muscle health determines autonomous activity and is a necessary factor for maintaining basic functions in the elderly. Age-related muscle atrophy is closely associated with metabolic diseases. Recently, an increasing number of studies have recognized the relationship between changes in adipose metabolism and distribution caused by aging and muscle health (2). There is an imbalance between muscle and adipose tissue (AT) mass with an increase in age, which is manifested as a decline in muscle mass and function starting at the age of 30 (3), decreasing by 6% every 10 years after middle age, and reducing by 30% at the age of 80 (4). The AT mass increases year by year from the age of 20 years, and reaches a peak at the age of 60–75 years (5), and the distribution shows a significant increase in visceral AT (6). This coexisting clinical state of sarcopenia and obesity is called obese sarcopenia, and its incidence increases with age (7).

Studies have found that degenerative changes and chronic diseases during aging could lead to low-grade inflammation of skeletal muscles, which is called “inflammatory aging” (8). A vicious age-related cycle has been established between AT and skeletal muscle inflammation. Chronic local inflammation (AT and skeletal muscle through paracrine/autocrine regulation) and systemic inflammation (through endocrine regulation) are the main mechanisms of sarcopenia (9). Pyroptosis is an inflammatory cell death pathway that depends on the activity of the cysteinyl aspartate-specific proteinase (caspase) family, and is characterized by the formation of micropores in the plasma membrane and the release of large amounts of pro-inflammatory cytokines. Pyroptosis is mainly mediated by inflammasomes; the NOD-like receptor thermal protein domain associated protein 3 (NLRP3) inflammasome is a typical member, and its complex include NLRP3, apoptosis-associated speck-like protein containing a CARD (ASC) and pro-Caspase1. After activation, active Caspase1 is formed to mature interleukin-1β and 18 (IL-1β/18), causing pyroptosis execution molecule gasdermin (GSDMD) cleavage, resulting in cell perforation, release of contents, and activation of nuclear factor kappa-B (NF-κB) and other inflammatory pathways (10). Studies have found that adipocyte pyroptosis is involved in the pathogenesis of obesity and related diseases (11), and the loss of key pyroptotic proteins leads to muscle atrophy (12).

Maintenance of skeletal muscle mass depends on the balance of protein metabolism. When protein decomposition is stronger than synthesis, muscle mass is lost. The mammalian target of rapamycin (mTOR) participates in skeletal muscle protein synthesis by regulating its downstream eukaryotic translation initiation factor 4E-bingding protein 1 (4E-BP1) (13), and the Forkhead transcription factor O1 (FoxO1)-mediated ubiquitin-proteasome system (UPS) is involved in the regulation of decomposition (14). The NLRP3 inflammasome mediates increased expression of inflammatory markers to promote protein decomposition, while increased IL-1β levels inhibit the mTOR pathway, and increased free fatty acid (FFA) levels caused by obesity inhibit protein synthesis (15). This suggests that pyroptosis plays an important role in the regulation of muscle mass. However, the regulatory effects of pyroptosis in sarcopenia remain unclear.

Weight-bearing running is a common exercise method that promotes muscle hypertrophy in rats (16, 17). Compared to ladder training, it can better control exercise intensity (18). Many studies have confirmed the effect of weight-bearing running training on delaying the process of aging-related muscle atrophy; however, from the perspective of inhibiting the chronic inflammatory state of skeletal muscle, few studies have explored the effect of weight-bearing training on relieving sarcopenia (19). Considering that fast-twitch muscles are more sensitive to aging and more easily promote hypertrophy by weight-bearing training, we chose the extensor digitorum longus (EDL) for further study. In this study, rats underwent weight-bearing running training with an increase in age to detect the levels of pyroptosis in EDL and AT and the metabolism of skeletal muscle proteins, to explore the role of pyroptosis in muscle atrophy, and to provide new targets for the prevention and treatment of age-related muscle atrophy.

## 2 Material and methods

### 2.1 Animals

Ninety male Sprague–Dawley rats (8-month-old, weighing 624.22 g± 61.63 g) were purchased from Sipeifu (Beijing) Biotechnology Co., Ltd. All the rats were reared at a temperature of 22 °C ± 2 °C, humidity of 50%–70%, 12-h light/dark cycle, and had *ad libitum* access to water and rodent chow (purchased from Beijing HFK Bioscience Co., Ltd), with three to four rats per cage (545 mm ∗ 395 mm ∗ 200 mm). After a week of acclimatization to the laboratory environment, 10 rats were randomly selected and sacrificed (N group). The remaining rats were randomly divided into two groups: a control group (C, *n* = 40) and a weight-bearing running group (R, *n* = 40). Rats in the C and R groups were sacrificed after 8, 16, 24, and 32 weeks (C/R8, C/R16, C/R24, and C/R32 groups, *n* = 10 for each group). All experimental protocols were approved by the Ethics Committee of the Sports Science Experiment Ethics Committee of the Beijing Sport University (Ref. No: 2019026A).

### 2.2 Training protocol

**Figure 1:**
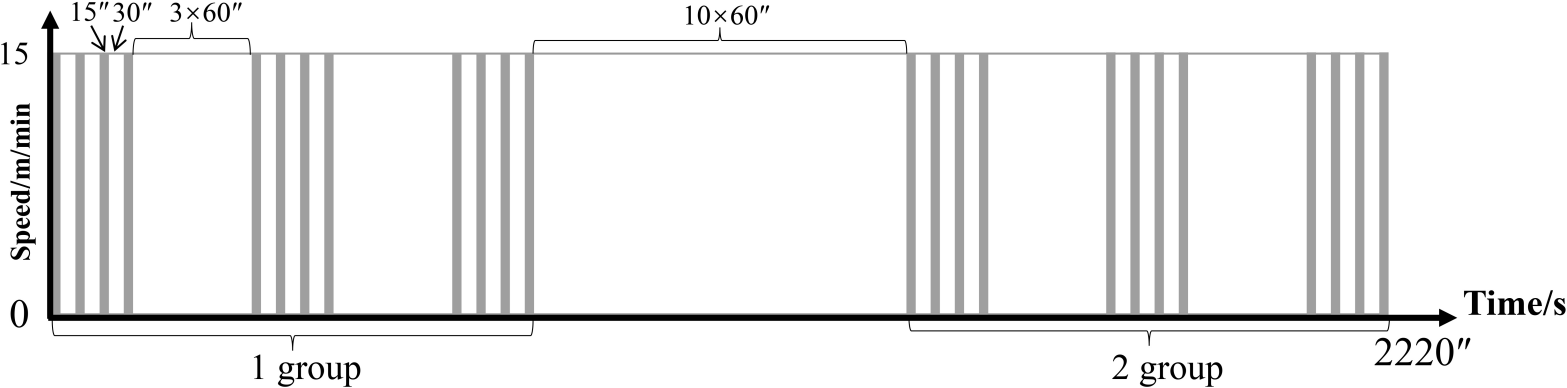
Training plan of the R group. The vertical axis represents speed (m/min) and the horizontal axis represents time (s).

The R group ware performed weight-bearing running training on a slope of 35°. Rats were outfitted with a steel ball-filled load-bearing vest (30% of body weight maximum load), rested for 30 s every 15 s running, four times as a group, rested for 3 min between groups, three groups as a cycle, and rested for 10 min to the next cycle, two cycles per day, as shown in **Figure 1** (19). Training was performed once every other day three times per week.

### 2.3 Sample collection

Rats were anesthetized with isoflurane in a small animal respiratory anesthesia system (Lab Animal Technology Develop Co., Beijing), and body composition was measured using dual-energy X-ray absorptiometry (DEXA) (Lunar Idxa) after 24 h of fasting every 8 weeks of training; then, blood was collected and stored at −80 °C, EDL and perirenal AT (visceral AT) were dissected and weighed, and histological morphology was observed. RNA levels were by PCR array and protein levels by Western blot.

### 2.4 Plasma lipid profiles

Plasma total cholesterol (TC), triglyceride (TG), low-density lipoprotein cholesterol (LDL-C), high-density lipoprotein cholesterol (HDL-C), and free fatty acid (FFA) levels were measured using an automatic biochemical analyzer (Beckman Coulter, AU480) using specific assay kits (Biosino Co., Ltd.).

### 2.5 Hematoxylin and eosin (H&E) staining

The EDL and AT were immersed in paraformaldehyde stationary solution (Cat. No. G1101, Servicebio, China) for 24 h, dehydrated with graded alcohol, replaced with xylene, soaked in paraffin, and embedded. Sections (4 μm) were dewaxed and stained with a HE Staining Kit (Cat. No. G1005; Servicebio, China), soaked in alcohol, and sealed. After imaging the sections using a microscope (Nikon, Japan), CaseViewer (3DHISTECH, Germany) was used to scan the muscle fiber and adipocyte morphology. Five fields of view were selected from the center and four corners of each slice and Image Pro Plus (IPP) 6.0 (Media Cybernetics, Inc. United States) was used by a third person to calculate muscle fiber and adipocyte cross-sectional area (CSA).

### 2.6 TdT-mediated dUTP-biotin nick end labeling (TUNEL) staining

EDL and AT paraffin sections were deparaffinized and rehydrated, retrieved with antigen, permeabilized and inactivated endogenous peroxidase, placed at room temperature to equilibrate, incubated in TUNEL reaction solution, mixed with Streptavidin-HRP and TBST, developed, counterstained in nuclei, dehydrated, dried briefly, and mounted with resin mounting medium. Five different fields of view were selected from the center and four corners of each slice, and the ImageJ software was used to count the percentage of TUNEL-positive cells.

### 2.7 Western blot

The total protein of the EDL was extracted and quantified using a protein assay kit (Thermo Fisher Scientific, USA). Protein samples were loaded onto 15 wells (20 μg total protein per well) of 4%–12% Bis-Tris or 3%–8% Tris Acetate gradient gels (NW04125/EA03755, Invitrogen, USA). After separation by electrophoresis, the proteins were transferred onto a nitrocellulose Regular Stack (IB23001, Invitrogen). Using Odyssey Blocking buffer (LI-COR) as the blocking reagent, the target proteins were blocked and probed overnight at 4°C with anti-mTOR (Abcam, ab134903), anti-p-mTOR (Cell Signaling Technology/CST, 5536T), anti-4E-BP1 (Proteintech, 60246-1-Ig), anti-p-4E-BP1 (CST, 2855), anti-FoxO1 (Santa, sc-374427), anti-p-FoxO1 (Abcam, ab131339), anti-ubiquitin (Santa, sc-8017), anti-NF-κB (CST, 8242), anti-ASC (Proteintech, 10500-1-AP), anti-Caspase1 (CST, 2225), and anti-β-tubulin (Proteintech, 10094-1-AP) antibodies. After washing three times (10 min per wash time) with Tris-buffered saline (TBS) containing Tween-20, the membranes were incubated with goat anti-rabbit or anti-mouse IgG secondary antibodies (LI-COR, 926-68071/926-32210) at 25°C for 1 h. Next, the membranes were washed twice with TBS and signals were detected using a near-infrared spectroscopy detection system (Odyssey CLX, LI-COR). All bands were analyzed semi-quantitatively using Image Studio Ver 5.2.

### 2.8 Pyroptosis PCR array

Total RNA of EDL was extracted using Trizol, checked for quality by UV absorption assay and agarose gel electrophoresis, and then synthesized into cDNA. The expression of 44 pyroptosis genes was detected using a PCR Array (wc-mRNA0275-R, WcGene Biotech, China). Gene expression was normalized to that of *GAPDH* and *UBB* using the 2-^△△^CT method. *P*-values were calculated by the t-test [false discovery rate (FDR) analysis showed no differentially expressed genes (DEGs)]. DEGs were screened using the following criteria: *p <*0.05 and fold change (FC) >1.5.

### 2.9 Real-time quantitative PCR analysis

Primer 5 software was used to design primers for the amplified genes, and the sequences were as follows: *Naip6*: forward primer: 5’-AGGGGGCTGTGGAAAGAAAC-3,’ reverse primer: 5’-GTGGTGAAGTCAACTCCCGT-3’; *Hmgb1*: forward primer: 5’-GCCCATTTTGGGTCACATGG-3,’ reverse primer: 5’-TGCAGGGTGTGTGGACAAAA-3.’ The WCGENE mRNA qPCR Quantification Kit (WC-SJH0002) was used. A two-step PCR amplification standard procedure was used: pre-denaturation at 95°C for 30 s, cycle amplification at 95°C for 5 s, 60°C for 30 s (40 cycles), and addition of a melting curve. *GAPDH* (forward primer: 5’-AACTCCCATTCCTCCACCTT-3,’ reverse primer: 5’-GAGGGCCTCTCTCTTGCTCT-3’) was used as an internal reference gene, and the relative expression of the target gene was calculated using the ^ΔΔ^Ct method.

### 2.10 Immunofluorescence (IF) staining

Paraffin sections of AT were washed with a gradient series of ethanol, rinsed with distilled water, and subjected to antigen retrieval. Next, the sections were quenched for autofluorescence, blocked with 5% bovine serum albumin, and then incubated with anti-NF-κB, ASC, GSDMD, Caspase-1, NLRP3 (Abcam, ab4207) antibodies overnight at 4°C. The next day, the sections were incubated with secondary antibodies (Servicebio, GB21303/926-32210) at room temperature for 50 min. DAPI counterstained for cell nuclei, mounted onto slides, and photographed under a confocal laser scanning microscope. Five different fields of view were selected from the center and four corners of each slice, IPP software (version 6.0) was used for analysis, and the integrated optical density (IOD) value of the selected area was measured (the image acquisition was performed by a third person blinded), indicating the intensity of immunopositivity.

### 2.11 Statistical analyses

Statistical analyses were performed using SPSS 22.0. All data are presented as the mean ± standard error of the mean. Student’s t-test or one-way ANOVA was used to determine significance. The Bonferroni method was used for the *post-hoc* test. The significance level was set at P <0.05.

## 3 Results

### 3.1 Body composition and degree of muscle atrophy

**Figure 2:**
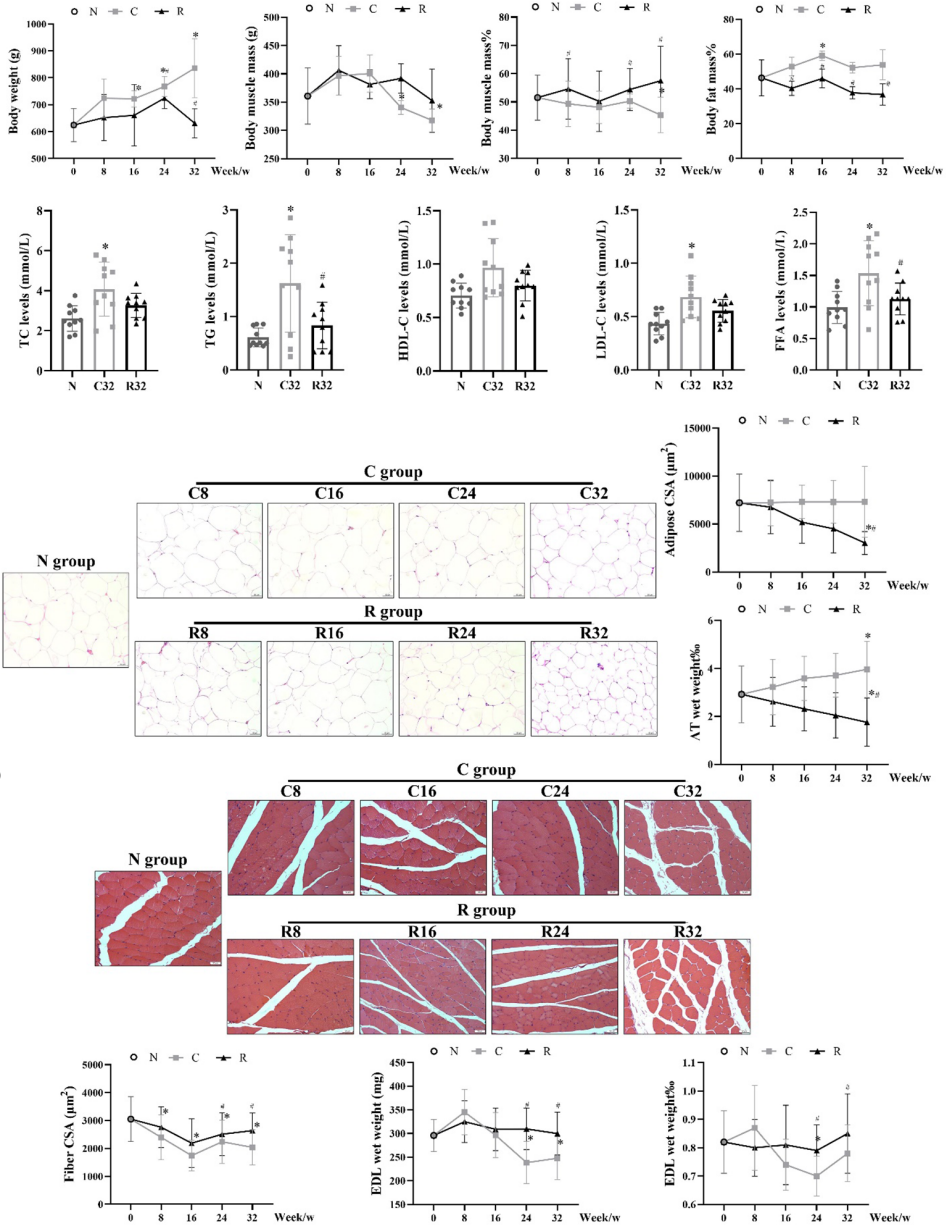
Body composition and degree of muscle atrophy in each rat group. **(A)** Body weight, muscle mass, muscle mass%, and fat mass% during the intervention (*n* = 10); **(B)** Levels of plasma lipid after the intervention (*n* = 10); **(C)** Adipocyte morphology, CSA, and AT wet weight% (*n* = 3); **(D)** Muscle fiber morphology, fiber CSA, and wet weight and percentage of EDL wet weight (EDL wet weight/total muscle mass) during the intervention (*n* = 3). ^*^Significant difference compared with the N group; ^#^Significant difference compared with the C group (*p <*0.05).

The body weights of the C group increased with age, and the body weights of the C16, C24, and C32 groups were significantly higher than those of the N group (*p <*0.05). The body weight of the R group was stable overall, and the weight of the R24 group was significantly higher than that of the N group, which was lower than that of the C24 group (*p <*0.05). The muscle mass of the C32 and C24 groups was significantly lower than that of the N group, and the percentage of muscle mass (muscle mass/body weight) in the C32 group was significantly lower than that in the N group (*p <*0.05); The percentage of LBM increased in the R group, and the percentage of LBM in the R8, R24, and R32 groups was significantly higher than that in the the corresponding C groups (*p <*0.05). The percentage of body fat mass (BFM) (BFM/body weight) of the C16 group was significantly higher than N group (*p <*0.05), and BFM of R8, R16, R24, and R43 groups were significantly higher than that in the corresponding C groups (*p <*0.05) (**Figure 2A**) (The absolute values of body muscle and fat as shown in **Figure S1**). After 32 weeks of intervention, the levels of serum TC, TG, LDL-C, and FFA in group C were significantly higher than those in N group (*p <*0.05), and the levels of TG and FFA in group R were significantly lower than those in group C (*p <*0.05) (**Figure 2B**). The adipocyte morphology of AT is shown in **Figure 2C**. The adipocyte CSA in the R32 group was significantly smaller than that in the C32 and N group (*p <*0.05), and the percentage of AT wet weight (AT wet weight/body weight) in the C32 group was significantly higher than that in the N group, while the R32 group was significantly lower than the C32 and N groups (*p <*0.05) (**Figure 2C**). The muscle fiber morphology of the EDL is shown in **Figure 2D**. Fiber CSA was significantly lower in the C group than in the N group, and CSA in the R24 and R32 groups was significantly higher than that in the C24 and C32 groups, respectively (*p <*0.05). The wet weight and percentage of EDL wet weight (EDL wet weight/total muscle mass) in the C24 group were significantly lower than those in the N group, and those in the R24 and R32 groups were significantly higher than those in the C24 and C32 groups (*p <*0.05) (**Figure 2D**).

### 3.2 The levels of pyroptosis and apoptosis in AT

**Figure 3:**
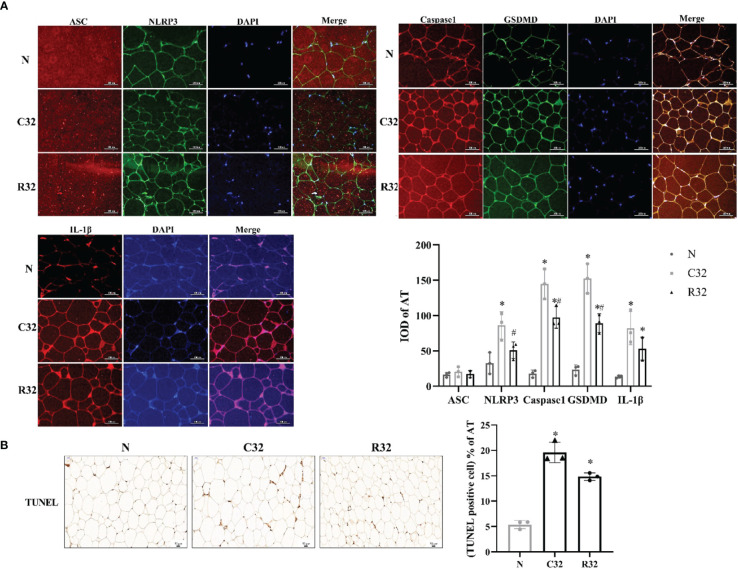
The level of pyroptosis and apoptosis of AT in rats after the intervention (*n* = 3). **(A)** The IF staining (×400) of ASC and NLRP3, Caspase1, and GSDMD, IL-1β, and the IOD of all these proteins in AT. **(B)** The TUNEL staining (×200) and the rate of apoptosis in AT. DAPI stained in blue, representing nuclei. Merge is a fusion graph. Nucleus stained with hematoxylin are blue. The positive apoptosis cells developed by DAB reagent have brown-yellow nucleus. ^*^Significant difference compared with N group; ^#^Significant difference compared with C group (*p <*0.05).

The IOD values of NLRP3, Caspase1, GSDMD, and IL-1β in the C32 group were higher than those in the N group (*p <*0.05). The IOD values of Caspase1, GSDMD, and IL-1β in the R32 group were also higher than those in the N group (*p <*0.05), and the IOD values of NLRP3, Caspase1, and GSDMD in the R32 group were lower than those in the C32 group (*p <*0.05) (**Figure 3A**). The apoptosis level of the C32 group was significantly higher than that of the N group, while decreased in the R32 group (*p <*0.05) (**Figure 3B**).

### 3.3 The levels of pyroptosis and apoptosis in EDL

**Figure 4:**
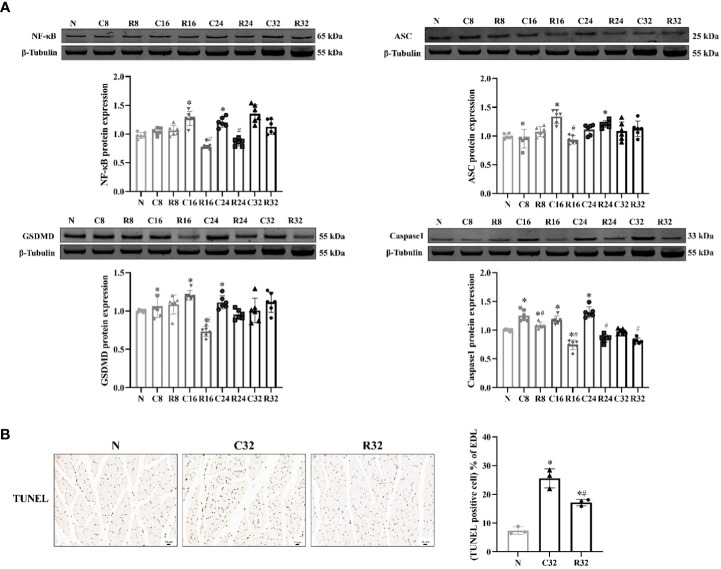
The levels of pyroptosis and apoptosis in the EDL during the intervention. **(A)** Protein expression levels of NF-κB, ASC, GSDMD, and Caspase1 (*n* = 6). **(B)** TUNEL staining (×200) and rate of apoptosis in the EDL (*n* = 3). The nucleus stained with hematoxylin are shown in blue. The apoptopic cells developed by DAB reagent had a brown-yellow nucleus. ^*^Significant difference compared with Group N; ^#^Significant difference compared with Group C (*p <*0.05).

The expression of NF-κB in the C16 and C24 groups was significantly higher than that in the N group, and that in the R16 and R24 groups was significantly lower than that in the C16 and R24 groups (*p <*0.05). ASC expression in the C16 group was significantly higher than that in the N group, and that in the R16 group was significantly lower than that in the C16 group (*p <*0.05). The expression of GSDMD in the C16 and C24 groups was significantly higher than that in the N group and that in the R16 group was significantly lower than that in the N and C16 groups (*p <*0.05). The expression of Caspase1 in the C8, C16, C24, and R8 groups was significantly higher than that in the N group, that in the R16 group was significantly lower than that in the N group, and that in the R group was lower than that in the C group (*p <*0.05) (**Figure 4A**). The apoptosis level of the C32 group was significantly higher than that of the N group but decreased in the R32 group (*p <*0.05) (**Figure 4B**).

### 3.4 The expression of pyroptosis-related genes of EDL

**Figure 5:**
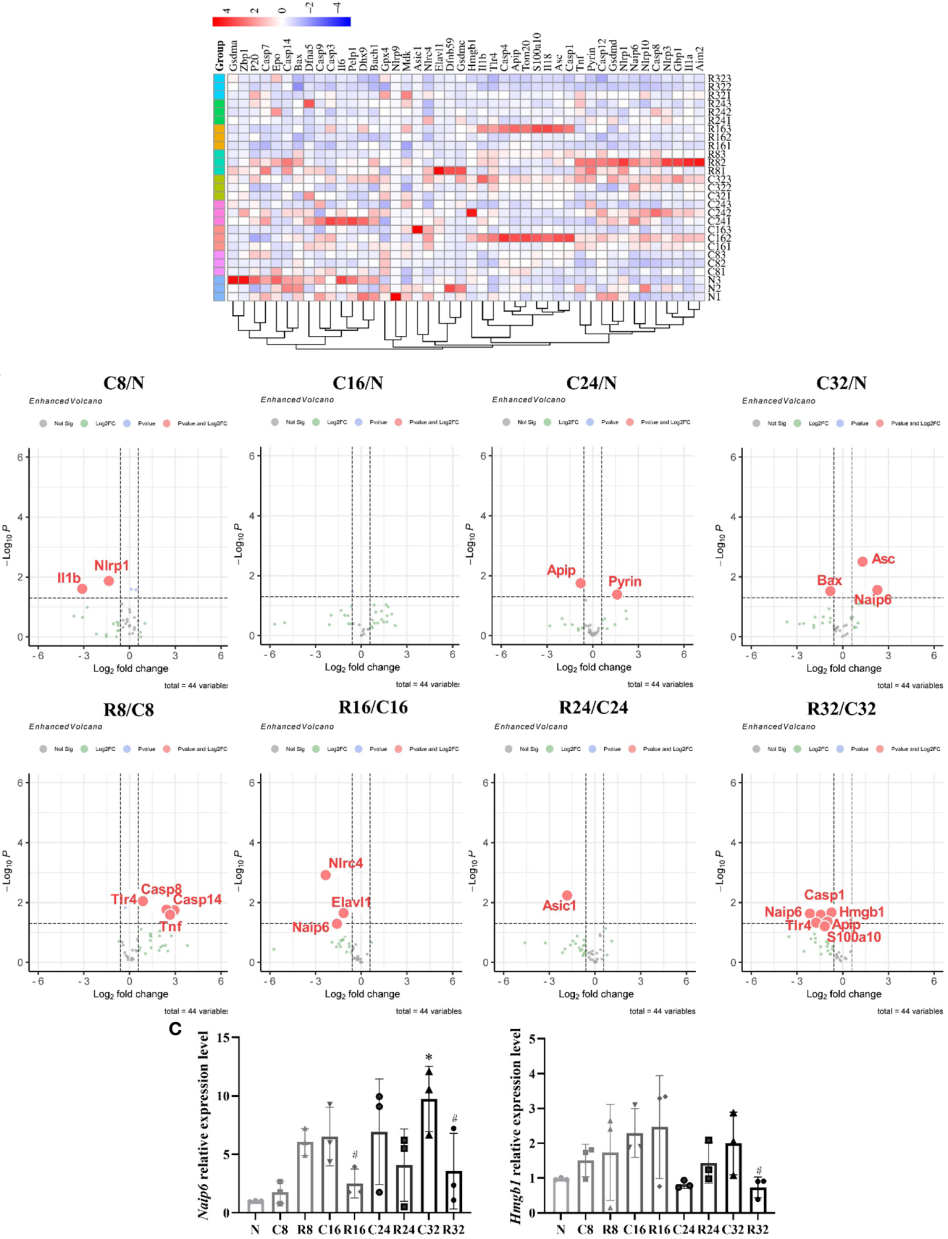
Expression of pyroptosis-related genes in the EDL during intervention (*n* = 3). **(A)** Cluster diagram of the 44 pyroptosis-related genes. The darker the color, the higher is the differential multiple. **(B)** Volcano plots of the DEGs in C8/N, C16/N, C24/N, C32/N, R8/C8, R16/C16, R24/C24, and R32/C32 groups. Red indicates upregulated differentially expressed genes and green indicates downregulated genes. **(C)** Expression levels of *Naip*6 and *Hmgb1* mRNA. ^*^Significant difference compared with the N group; ^#^Significant difference compared with the C group (*p <*0.05).

With the increase of age, the number of upregulated pyroptotic genes increased; with the prolongation of training time, the number of upregulated pyroptotic genes decreased (**Figure 5A**). The FC and *P-*values of the DEGs are shown in volcano plots (**Figure 5B**). The downregulated gene in the C8/N group was *Nlrp1*; there were no DEGs in the C16/N group; the upregulated differential gene in the C24/N group was *Pyrin*; the upregulated gene in the C32/N group was *Naip6*; the upregulated genes in the R8/C8 group were *Casp8*, *Casp14*, and *Tnf*; the downregulated genes in the R16/C16 group were *Naip6*, *Nlrc4*, and *Elavl1*; and the downregulated gene in the R24/C24 group was *Asic1*; The downregulated genes in R32/C32 group were *Naip6*, *Casp1*, *Tlr4*, *Hmgb1* and *Apip*. *Naip6* and *Hmgb1* were selected for qPCR validation. The results showed that the expression of *Naip* mRNA in the C32 group was significantly higher than that in the N group, the R16 and R32 groups were significantly higher than those in the corresponding C group (*p <*0.05), and the expression of *Hmgb1* mRNA in the R32 group was significantly higher than that in the C32 group (*p <*0.05) (**Figure 5C**), which was consistent with the array results.

### 3.5 Synthesis and decomposition of skeletal muscle protein of EDL

**Figure 6:**
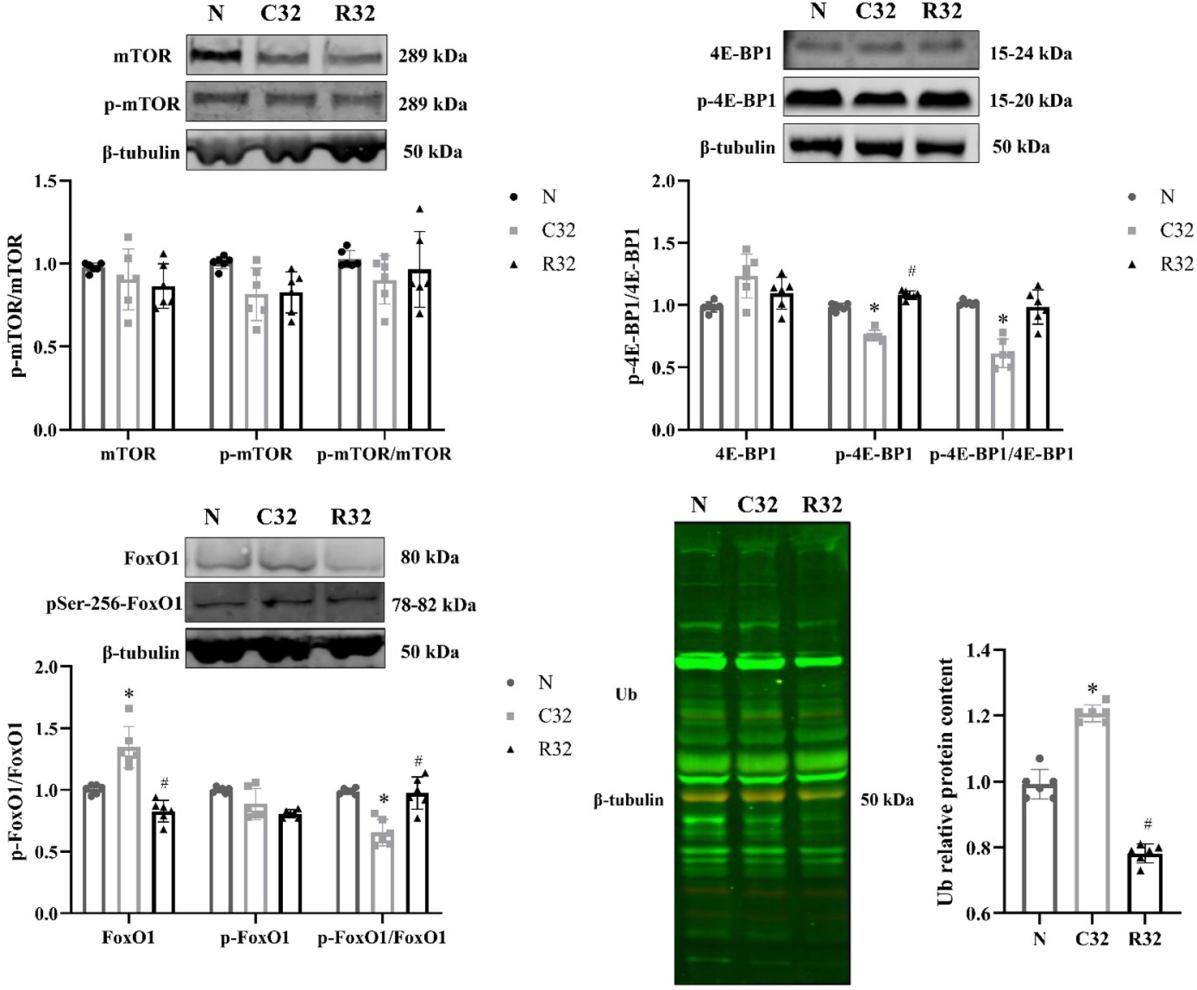
Synthesis and decomposition of skeletal muscle proteins in the EDL after 32 weeks of intervention (*n* = 6). Protein expression of mTOR and p-mTOR; ratio of p-mTOR/mTOR; protein expression of 4E-BP1 and p-4E-BP1; ratio of p-4E-BP1/4E-BP1; protein expression of FoxO1 and p-FoxO1; the ratio of p-FoxO1/FoxO1; and protein expression of Ub. ^*^Significant difference compared with the N group; ^#^Significant difference compared with the C group (*p <*0.05).

mTOR, 4E-BP1, and their phosphorylation levels were used as indicators of protein synthesis. There were no significant differences in mTOR and phospho-mTOR (p-mTOR) protein expression and p-mTOR/mTOR ratio in each group; p-4E-BP1 protein expression and p-4E-BP1/4E-BP1 ratio in the C32 group were significantly lower than those in the N group, and p-4E-BP1 protein expression in the R32 group was significantly higher than that in the C group (*p <*0.05). FoxO1 phosphorylation and protein ubiquitin (Ub) labeling levels reflect protein decomposition. FoxO1 and Ub protein expression in the C32 group was significantly higher than that in the N group, the R32 group was significantly lower than that in the C32 group, the ratio of p-FoxO1/FoxO1 in the C32 group was significantly lower than that in the N group, and the R32 group was significantly higher than that in the C32 group (*p <*0.05) (**Figure 6**).

## 4 Discussion

### 4.1 Weight-bearing training alleviates the process of age-related muscle atrophy and reduces adipose accumulation

As non-drug therapy, weight-bearing training can reverse muscle atrophy or cause adaptive hypertrophy by increasing muscle mass and volume and improving muscle strength (20). In a meta-analysis of 49 studies, including a total of 1,328 subjects aged over 50 years, the average increase in skeletal muscle mass was 1.1 kg after an average of 20.5 weeks of weight-bearing training (2–3 times/week) (21). Therefore, the role of weight-bearing training in the prevention and treatment of age-related muscle atrophy has been widely confirmed. The weight bearing treadmill training cycle generally lasts more than 10 weeks for rats. This exercise method accurately controls the exercise load and is convenient for repeated experiments (22). For the elderly, attention should be paid to avoid skeletal muscle damage caused by excessive exercise intensity and volume. Therefore, this study referred to the most suitable weight-bearing running program obtained by Weng et al. (2013) (23). intervention program. Our results showed that body weight, body fat percentage, AT wet weight, adipocyte CSA, and plasma lipid levels were reduced, but muscle mass and its percentage, EDL wet weight, and FCSA were increased in aged rats after 32 weeks of weight-bearing running training.

### 4.2 Weight-bearing training promotes protein synthesis and inhibits decomposition in muscle with an increase in age

The promotion of protein synthesis is the general mechanism through which exercise promotes muscle hypertrophy. Protein synthesis is a key factor affecting skeletal muscle mass in healthy organisms. However, in models of atrophy, inhibition of proteolysis may play a more critical role in maintaining or increasing muscle mass (24). Insulin-like growth factor (IGF) is activated by exercise and promotes mTOR phosphorylation, further phosphorylates 4E-BPl, and causes eukaryotic translation initiation factor eukaryotic translation initiation factor 4E (eIF-4E) to decompose to initiate the protein translation process (25). In this study, the levels of p-4E-BP1 and p-4E-BP1/4E-BP1 decreased with increasing age, which was alleviated by p-4E-BP1.

FoxO1 is a key nuclear transcription factor that regulates muscle mass, and its biological activity is lost when it exits the nucleus after phosphorylation. Proteins that are degraded by protease are marked by Ub, which constitutes the main link of the UPS in protein decomposition. Our study showed that the levels of FoxO1 and Ub increased, and the ratio of p-FoxO1/FoxO1 decreased with aging, which was reversed by weight-bearing training.

### 4.3 Weight-bearing training inhibits pyroptosis and improves body composition of rats with an increase in age

The relationship between exercise and the inflammatory response is complex. Moderate-intensity exercise reduces inflammation through the expression of hormones with immunomodulatory effects and toll-like receptors (TLR). The study found that the levels of plasma IL-6, leptin, and resistin decreased in sedentary women with an average age of 63 years after 16 weeks of weight-bearing training (26), skeletal muscle protein synthesis improve, and body fat mass was reduced by inhibiting the release of pro-inflammatory factors in patients with sarcopenic obesity by weight-bearing training (27).

Pyroptosis is an inflammatory programmed cell death pathway, and an important natural immune response that induces muscle atrophy. Upregulation of NF-κB signaling may enhance inflammasome expression during aging (28), and myotube atrophy can be caused by pyroptosis induced by lipopolysaccharide (LPS) (29). The key pyroptotic protein defect in muscular dystrophy mice alleviates atrophy and improves muscle function (30), and the inhibition of Caspase1/GSDMD/IL-1β pathways ameliorates skeletal muscle pathology in rats with impaired glucose tolerance induced by a high-fat diet (31). These studies suggest that pyroptosis can be inhibited to alleviate age-related muscle atrophy. In this study, the protein expression of NF-κB, ASC, GSDMD, and Caspase1 significantly decreased, and the apoptosis rate also decreased after intervention. The downregulation of pyroptosis-related genes gradually increased with increasing training time. Nucleotide-binding oligomerization domain-like receptor family apoptosis inhibitory protein 6 (*Naip6*) was upregulated in the C32/N group but downregulated in the R16/C16 and R32/C32 groups. Naip is closely related to the pathogenesis of spinal muscle atrophy, and Gene Ontology (GO) analysis of the ubiquitin protein transferase activity of Naip involved in apoptosis (32). The R32/C32 group down-regulated gene high mobility group box 1 (*Hmgb1*), which was also closely related to the regulation of muscle atrophy. As the main receptor of Hmgb1, TLR4 and its downstream NF-κB signaling pathways promote muscle protein decomposition(33). Hmgb1 is involved in denervation-induced muscle atrophy, which may be mediated by transforming growth factor β (TGF-β)/Hmgb1 activation of the proteolytic pathway (34). Hmgb1 attracts more attention in sports science in recent years, which is considered as “alarmin: that induces a systemic sterile inflammatory state during acute high-intensity exercise. In contrast, Hmgb1 expression is inhibited and health is improved by long-term regular exercise training (35).

In this study, weight-bearing running training not only inhibited age-related muscle atrophy but also reduced the expression of key proteins involved in pyroptosis in AT. It has been reported that the immune response is the most relevant feature of aging in AT, which can be observed in middle-aged individuals (36). Pro-inflammatory cytokines secreted from AT are considered major elements of systemic chronic low-grade inflammation during aging. AT produces 30% IL-6 in the circulatory system, which is a pro-inflammatory factor, and the level of IL-6 in visceral AT is higher than that in subcutaneous AT (37). We found that blood lipid levels increased with age, and it has been proven that the NLRP3 inflammasome is activated by high levels of LDL-C and TC, while the deletion of NLRP3 prevents lipid deposition in obesity (38). It has been reported that the activation of NLRP3 is inhibited in the AT of obese individuals after 8 weeks of moderate-intensity treadmill training (39). Exercise promotes the release of peroxisome proliferator-activated receptor-gamma coactivator-1 α (PGC1α) and AMP-activated protein kinase (AMPK) from the skeletal muscle into the blood, participating in the inhibition of AT inflammation and improving lipid metabolism by regulating macrophage activity (40).

## 5 Conclusions

Age-related adipose accumulation and muscle loss were accompanied by increased levels of pyroptosis in the EDL and AT, but could be reversed by weight-bearing training, which could inhibit the expression of pyroptosis-related genes and key proteins, alleviating the imbalance of protein metabolism.

## Data availability statement

The datasets presented in this study can be found in online repositories. The names of the repository/repositories and accession number(s) can be found in the article/**Supplementary Material**.

## Ethics statement

The animal study was reviewed and approved by Ethics Committee of Sports Science Experiment Ethics Committee of Beijing Sport University.

## Author contributions

PF, ST, LY, and FM performed most of the study and conducted experiments. PF wrote the original manuscript. LG reviewed the data, revised the manuscript. All authors listed have made a substantial, direct, and intellectual contribution to the work and approved it for publication.
