# Supplementary material for: Weight bearing training alleviates muscle atrophy and pyroptosis of middle-aged rats
Source: Front Endocrinol (Lausanne). 2023 Aug 30;14:1202686. doi: 10.3389/fendo.2023.1202686 (PMC10499618; doi:10.3389/fendo.2023.1202686)
Supplement: Supplementary file 2 [file Image_1.pdf]

Supplementary Materials

Lean Body Mass and Fat Mass

The lean body mass of R24 group were significantly higher than N group ($p < 0.05$) (Figure S1A). The body fat mass of C16, C24 and C32 groups were significantly higher than N group ($p < 0.05$), and R8, R16, R24, R43 groups were significantly higher than their corresponding C groups ($p < 0.05$) (Figure S1B).

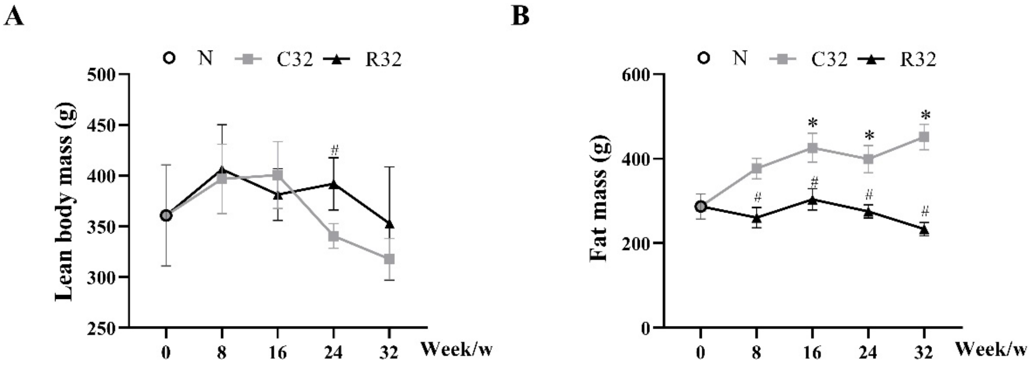

Figure S1 Lean body mass and body fat mass of rats. (A) Lean body mass; (B) Fat mass (*n*=10). *Significant difference compared with N group; #Significant difference compared with C group ($p < 0.05$).
